# Appropriate use of plasma glucose tests for diagnosis of diabetes mellitus in Ibadan, Nigeria

**DOI:** 10.4102/ajlm.v11i1.1433

**Published:** 2022-04-29

**Authors:** Modupe A. Kuti, Olabisi T. Bamidele, Chioma T. Udeh, Bola J. Eseile, Olajumoke A. Ogundeji

**Affiliations:** 1 Department of Chemical Pathology, Faculty of Basic Medical Sciences, College of Medicine, University of Ibadan, Ibadan, Nigeria; 2 Department of Chemical Pathology, University College Hospital, Ibadan, Nigeria

**Keywords:** diabetes mellitus, glucose, utilisation management, laboratory, guidelines, fasting, postprandial, random

## Abstract

**Background:**

Diabetes mellitus is a growing epidemic in Africa. Its diagnosis relies exclusively on laboratory evidence, which differs based on clinical circumstances.

**Objective:**

The study described the appropriateness of plasma glucose test requests per the American Diabetes Association criteria.

**Methods:**

We reviewed the plasma glucose test requests received by the chemical pathology laboratory of the University College Hospital, Ibadan, Nigeria between June 2018 and November 2018. The American Diabetes Association diabetes diagnostic criteria were used to define the appropriateness of test requests and determine the potential for ill-informed clinical decisions.

**Results:**

Four hundred and twenty-three requisition forms were included, with the majority from the medical wards/clinics (72.3%); the most frequent reason for a plasma glucose test was systemic hypertension (28.6%). Fasting plasma glucose was most requested (254; 60.0%). One hundred and sixteen (27.4%) requests were potentially inappropriate, with the 2-h postprandial plasma glucose (2hPPG) test requests (83; 71.6%) being the most inappropriate. The difference in the proportion of inappropriate requests was not statistically significantly between medical or surgical wards/clinics (Odds ratio 1.131, 95% confidence interval 0.709–1.803, *p* = 0.605). Inappropriate requests in six cases may have triggered inappropriate action.

**Conclusion:**

A third of the glucose tests requested for querying diabetes mellitus may have been inappropriate. Results of such testing may trigger inappropriate clinical action. To improve the quality of care and for economic reasons, laboratories should have programmes to improve the appropriate use of their services.

## Introduction

The socio-economic burden of a diabetes mellitus (DM) diagnosis is substantial for persons in low and middle-income countries relative to persons in higher-income countries.^1^ The absence of robust national health insurance schemes in low and middle-income countries means that this burden is borne mainly by patients and their families. The total healthcare cost of persons with DM has been estimated to be about four times higher than for persons with normal glucose tolerance.^2^ This cost includes those incurred from admissions, outpatient visits, laboratory tests, medication, and treatment trips, as well as productivity losses by patients and caregivers. The rapidly rising prevalence of DM imposes an increasing significant strain on fragile national health systems,which are already buckling under a huge infectious disease burden. Estimates of DM prevalence from urban populations in Kenya, Cameroon, and South Africa range between 10% and 12%.^3,4,5^ A recent Lancet commission on the burden of DM in sub-Saharan Africa reported that DM and its complications are costly to patients and that national health systems are largely unable to cope with the current burden of the disease.^6^

One of the fundamental requirements for appropriate public health response to this epidemic is accurate diagnosis of DM. The diagnosis of DM relies exclusively on biochemical evidence of a specific degree of glucose intolerance. The current accepted diagnostic criteria for DM in the non-pregnant adult were initially published by the American Diabetes Association in 1998 and were adopted by the World Health Organization in 1999.^7,8^ The laboratory tests referenced in the criteria are fasting plasma glucose (FPG), 2-h post-load glucose (2hPLG) during an oral glucose tolerance test (OGTT), and random plasma glucose (RPG). In 2010, glycated haemoglobin was listed as a further diagnostic criterion.^9^ Other than glycated haemoglobin, all the glucose criteria require that specific patient preparation steps or clinical symptoms be present. The FPG sample is collected after an overnight fast of at least 8 h, while the 2hPLG sample is obtained 2 h after the patient consumes a standard glucose load of 75 g of anhydrous glucose. For RPG to be used as a DM diagnostic criterion, the presence of either classic symptoms of DM or hyperglycaemic crisis is required. This suggests that RPG is not recommended for screening of the asymptomatic person and 2hPLG should not be used interchangeably with 2-h postprandial plasma glucose (2hPPG). The latter test is performed on a sample taken after the consumption of the individual’s regular diet, which will vary significantly from person to person. Two-hour postprandial plasma glucose has utility in assessing glycaemic control in persons with known DM.^10^

The study aimed to determine the appropriateness of the glucose test requests per the American Diabetes Association DM diagnosis criteria.^11^ Furthermore, for tests that were ordered contrary to recommended guidelines (inappropriate tests), we examined the potential for consequent inappropriate clinical action based on the result of such tests.

## Methods

### Ethical considerations

Ethical approval was obtained from the University of Ibadan/University College Hospital Ethics Committee with a National Health Research Ethics Committee NHREC/05/01/2008a. The ethical committee assigned number for the study was UI/EC/19/0630. This was an analysis of secondary data and did not retrieve any patient-identifiable information or involve contact with any human participants. Patient consent was therefore not required. All glucose results were anonymised from patient details and study numbers accessible only to the researchers were used during analysis.

### Study setting

The University College Hospital, Ibadan is a public tertiary hospital located in an urban setting in the South Western region of Nigeria. The hospital is served by a Chemical Pathology laboratory that receives about 33 000 samples annually, including blood, urine, and cerebrospinal, ascitic and pleural fluids. Tests provided include those assessing for renal, liver, metabolic, endocrine, and neoplastic diseases. For a non-pregnant adult, the laboratory offers two stand-alone glucose tests – FPG and RPG – as well as two glucose profiles – FPG/2hPPG and FPG/2hPLG.

### Study design

This cross-sectional study reviewed all request forms from the wards/clinics of the hospital from June and November 2018 for plasma glucose tests. To be included in this study, the section for clinical information was required to contain information about the patient such as presenting symptoms, signs, working diagnosis, or treatment. Any form with no clinical information or with clear information that the patient was previously diagnosed as having DM was excluded.

### Definition of test request inappropriateness

The appropriateness of the tests was defined using the American Diabetes Association DM diagnostic criteria (Box 1).^12^ The following test requests were deemed as probably inappropriate requests for DM diagnosis:

- All 2hPPGtest requests.
- All RPG test requests in which the clinical details on the requisition form included none of the classic symptoms of DM (polyuria, polydipsia, and weight loss).

BOX 1 Criteria for the diagnosis of diabetes mellitus by the American Diabetes Association (2018).

**Diagnostic Criteria**

FPG ≥ 7.0 mmol/L. Fasting is defined as no caloric intake for at least 8 h.†

2hPLG glucose ≥ 11.1 mmol/L during an OGTT. The test should be performed as described by the WHO, using a glucose load containing the equivalent of 75 g anhydrous glucose dissolved in water

A1C ≥ 6.5%. The test should be performed in a laboratory using a method that is NGSP certified and standardised to the DCCT assay.†

In a patient with classic symptoms of hyperglycaemia or hyperglycaemic crisis, RPG ≥ 11.1 mmol/L.

*Source*: American Diabetes Association. Classification and diagnosis of diabetes: Standards of medical care in Diabetes 2018. Diabetes Care. 2018;41(Suppl 1):S13–S27. https://doi.org/10.2337/dc18-S002

FPG, fasting plasma glucose; 2hPLG, 2-h post-load glucose; OGTT, oral glucose tolerance test; WHO, World Health Organization; A1C, Haemoglobin A1C; NGSP, National Glycohaemoglobin Standardization Programme; DCCT, Diabetes Complications and Control Trial; RPG, random plasma glucose.

†, In the absence of unequivocal hyperglycaemia, criteria 1–3 should be confirmed by repeat testing.

The potential for inappropriate clinical action was deemed to be present if:

- The result of an inappropriately requested RPG exceeded 11.1 mmol/L, and/or
- The FPG was < 7.1 mmol/L and the 2hPPG was > 11.1 mmol/L.

### Data analysis

Statistical analysis was performed using IBM^®^ Statistical Package for Social Sciences (SPSS^®^) version 25 (2017, IBM Corp, Armonk, New York, United States). Proportions are presented as numbers (percent) and were compared using the chi-square test. Results were considered significant if *p* was 0.05 or less, with a 95% confidence interval.

## Results

Four hundred and twenty-three request forms satisfied the inclusion criteria. Medical outpatient clinics and wards accounted for 306 (72.3%) of these requests. Overall, the most common reason for a glucose test request was to evaluate systemic hypertension (Table 1). The surgical clinics and wards most commonly requested a glucose test for evaluating neoplastic disease (benign or malignant). A one-off FPG was the most requested glucose test in both specialities, accounting for 254 (60.0%) of all requests (Table 2). The usage pattern for the different tests/profiles was significantly different between both specialities (*X*^2^ = 138.911 [*p* < 0.001]); the surgical specialities requested more OGTT, mostly for postnatal assessment of persons with previous gestational DM.

**TABLE 1:** Clinical information on requisition forms received in the Chemical Pathology Laboratory, University College Hospital, Ibadan Nigeria (June 2018 – November 2018).

Clinical Information	Total	
	Number	Percent
Hypertension	121	28.6
Diabetes mellitus screen	35	8.3
Neoplasm related†	27	6.4
Infection related‡	23	5.4
Medical screen	22	5.2
Other vascular disease related§	20	4.7
Postnatal assessment	19	4.5
Peripheral neuropathy	18	4.3
Other endocrine disease¶	18	4.3
Polyuria	13	3.1
Preoperative assessment	14	3.3
Others	93	22.0

† , Neoplasm related – includes breast cancer, benign prostatic hypertrophy, uterine fibroids;

‡ , Infection related – includes recurrent vaginal infections, recurrent urinary tract infections, dental abscess, and furunculosis;

§ , Other vascular disease-related – includes stroke, arrhythmias, heart failure, and coronary syndrome;

¶ , Other endocrine disease-related – includes thyrotoxicosis, Plummer’s disease.

**TABLE 2:** Distribution of glucose requests by speciality of source ward/clinics of the University College Hospital, Ibadan, Nigeria (June 2018 – November 2018).

Glucose profile	Total		Medical		Surgical		*χ* ^2^	*P*
	*n*	%	*n*	%	*n*	%		
FPG alone	254	60.0	209	68.3	45	38.5	138.911	< 0.001
FPG and 2hPPG	83	19.6	60	19.6	23	19.7		
RPG	33	7.8	22	7.2	11	9.4		
OGTT	53	12.5	15	4.9	38	32.5		

**Total**	**423**	**100.0**	**306**	**100.0**	**117**	**100.0**	-	-

FPG, fasting plasma glucose; 2hPPG, 2-h postprandial plasma glucose; RPG, random plasma glucose; OGTT, oral glucose tolerance test.

The FPG level was normal (< 5.5 mmol/L) in 77.7% (303) of cases, impaired (5.5 mmol/L – 6.9 mmol/L) in 15.1% (*n* = 59), and diabetic (≥ 7.0 mmol/L) in 7.2% (*n* = 28). The glucose level was < 11.1 mmol/L for 88% (*n* = 73) 2hPPG, 88.7% (*n* = 47) 2-h OGTT and 100% (*n* = 33) RPG requests.

One hundred and sixteen (27.4%) of the test requests were potentially inappropriate, comprising 83 (71.6%) requests for FPG/2hPPG and 33 (28.4%) requests for RPG (Table 3). All of the OGTT requests were appropriate. Inappropriate test requests from the medical wards and clinics (*n* = 82; 70.7%) were higher than those from the surgical wards and clinics (48 tests; *p* = –0.629) (Table 2). Of the 83 inappropriately requested FPG/2hPPG, the FPG was < 7 mmol/L while the 2hPPG was > 11.1 mmol/L in 6 (7.2%) cases. The FPG and accompanying 2hPPG were < 7 mmol/L and < 11.1 mmol/L in 71 (85.5%) cases. Fasting plasma glucose > 7 mmol/L/2hPPG < 11.1 mmol/L was seen in one case and FPG > 7 mmol/L/2hPPG > 11.1 mmol/L was seen in another five cases.

**TABLE 3:** Clinical information against inappropriately requested glucose tests in the Chemical Pathology Laboratory, University College Hospital, Ibadan Nigeria (June 2018 – November 2018).

Clinical Information	RPG		FPG/2hPPG	
	*n*	%	*n*	%
Hypertension	13	32.5	27	67.5
Infection related†	3	18.8	13	81.2
Neoplasm related‡	1	12.5	7	87.5
Other vascular disease related§	3	37.5	5	63.5
Diabetes mellitus screen	2	33.3	4	66.7
Medical screen	4	80.0	1	20.0
Musculoskeletal disease related¶	1	12.5	7	87.5
Other clinical information	7	26.9	19	73.1

**Total**	**33**	**100.0**	**83**	**100.0**

RPG, random plasma glucose; FPG, fasting plasma glucose; 2hPPG, 2h postprandial plasma glucose;

† , includes recurrent vaginal infections, recurrent urinary tract infections, dental abscess, and furunculosis;

‡ , includes breast cancer, benign prostatic hypertrophy, uterine fibroids;

§ , includes stroke, arrhythmias, heart failure, and coronary syndrome;

¶ , includes lower back pain, muscle spasm.

## Discussion

Despite the annual publication of the American Diabetes Association standards for the laboratory diagnosis of DM for over 20 years, this study provides evidence that there exists a need to increase the uptake of its recommendations in routine practice in our setting. Nearly one out of every three requests for glucose tests, in our setting, was found to be potentially inappropriate. Inappropriate requests were mostly due to requests for 2hPPG as a DM screening test. Reports from both Nigeria and Cameroon indicate that primary care physicians, who represent the bulk of physicians providing DM screening and care, have poor knowledge of the diabetes clinical guideline criteria.^13,14^ Over 70% of the participants in both studies were not reliably diagnosed with DM using the requested glucose tests.^13,14^

The primary consequence of an inappropriate glucose test is the waste of the patient’s money for an unnecessary test. The wastage is particularly costly in a region where most patients are uninsured and thus pay out of pocket.^15^ Money spent on unnecessary testing may reduce money for necessary treatment. There is also a cumulative cost to the health systems. Glucose tests are relatively inexpensive, however, increased requests over time will drain laboratory resources.^16, 17^

A further consequence of inappropriate testing is a false negative test result. As observed in this study, the majority of the requests for RPG returned values below a ‘diagnostic’ threshold. There is no guarantee that the screened patients were all glucose tolerant. Reports suggest that the sensitivity of an RPG value greater than or equal to 7 mmol/L for identifying asymptomatic persons with DM may be less than 70%.^18,19^ These reports concluded that it would be inappropriate to use the RPG test alone as a DM screening test. Thus, an additional test is required for definitive diagnosis of DM, thereby increasing costs incurred by patients.

We examined whether the requests from surgical wards and clinics were more likely to be inappropriate compared to those from medical wards and clinics. Reports suggest that certain practices by surgeons may increase the likelihood of inappropriate test requests. Charani et al.^20^ reported that surgeons frequently left care decisions perceived as non-surgical to junior team members. This practice is reportedly associated with an increased likelihood of inappropriate laboratory test requests.^21,22^ Our study, however, did not observe a significant difference in rates of inappropriate test requests from the surgical wards and clinics compared to the medical ones. A possible explanation for this observed lack of significant difference in inappropriate test requests between both wards and clinics in this study might be due to, the much higher number of test requests from the medical areas consequently increasing the number of inappropriate requests. This may thus cancel out the effect of the delegation of duties within the surgical areas.

We also note that, although a 2hPPG > 11.1 mmol/L may be suggestive of the presence of DM, it may not be used as conclusive evidence of the disease, as current criteria for DM diagnosis do not include it. Glycaemia > 11.1 mmol/L may be observed as part of a physiologic stress response.^23^ The appropriate response, therefore, to such a result should be an evaluation for DM using tests listed in the currently accepted criteria. Any other clinical course, such as commencing treatment or re-ordering the same test, would be inappropriate.

The present study has demonstrated that a significant percentage of requests for plasma glucose, a routine and frequently test, may be inappropriate. Similarly, estimates from a teaching hospital in Austria suggest that as many as 60% – 70% of high throughput tests such as potassium and lactate dehydrogenase may have been ordered inappropriately or in clinical situations where they had only minor relevance.^24^ Depending on the criteria used, estimates of request inappropriateness from the United States range between 5% and 95% of tests.^25^ We have also shown here the potential for inappropriate clinical action following an inappropriate laboraory test requests. A 20-year review of malpractice claims in insurance companies that provide cover for patients in over 40 academic and non-academic hospitals in the United States found 181 claims that involved diagnostic errors and resulted in harm to the patient. Over 50% of these claims were attributable to a failure to order an appropriate test.^26^ Inappropriate test usage wastes resources and may result in actual harm to the patient.

Predictably, a system to manage physician test utilisation has value for patients, the physicians, and the health care system, with significant positive yields in quality of care and economic savings.^27,28^ Such a system should be characterised by an understanding of the multitude of factors that influence test requisition. These range from diagnostic, therapeutic/prognostic, patient-related, physician-related and policy/organisation factors.^29^ Depending on the specific intention of such a management programme, a wide range of approaches is available.^30,31^ These approaches include the use of utilisation audits, as in the current study, analytical algorithms, test guidelines, formularies and, where applicable, clinical decision support systems and changes to computerised provider order entry. Implementing utilisation management initiatives requires the collaboration of key stakeholders, including pathologists, laboratorians and other professionals who contribute to the health care process.^28^ Pathologists, whose background training enables them to observe testing behaviour patterns, suggest alternatives, manage testing algorithms and provide interpretative services, could show leadership in this regard.^28,32,33^

### Limitations

The study was limited by its reliance on the information included in the submitted requisition forms. This information may not have correctly reflected the entire clinical status of the patient being evaluated.

### Conclusion

In conclusion, we have demonstrated the suboptimal usage of glucose tests in DM screening, with the speciality of the requesting physician as a potential factor. This provides evidence that laboratories should engage in programmes, such as standard diagnosis awareness campaigns, directed at improving the appropriate use of their services.
